# AI-assisted rational decision-making

**DOI:** 10.1007/s11229-026-05528-7

**Published:** 2026-03-23

**Authors:** Daniel Villiger

**Affiliations:** 1 Institute of Philosophy, University of Zurich, Zollikerstrasse 117, Zürich, 8008 Switzerland; 2 Uehiro Oxford Institute, University of Oxford, Oxford, UK

**Keywords:** AI, Rational choice, Hard choices, Parity, Transformative experience

## Abstract

AI has become a common assistant for making choices, from minor to major ones. It can inform our beliefs relevant to a decision by both helping us to find existing information and generating new information. But in what ways and to what extent is AI useful when making a rational decision? The present paper provides answers to this question for three different types of choices: easy choices, hard choices, and transformative choices. In easy choices, where the rational action is, in principle, straightforward, AI can make the decision-making process more efficient and accurate, increasing derived value (at least in the long-term). In hard choices, where options are on a par, AI can help us when we commit to an option by assisting us in the creation process of new will-based reasons. In transformative choices, where we cannot, even in principle, know by ourselves which option maximizes expected value, AI cannot fill the epistemic or metaphysical gap characteristic of such choices, and therefore cannot enable rational decision-making. Overall, the analysis shows that if the values of our options do not already allow us to determine the rational choice without AI, its assistance does not change that.

## Introduction

At least since the public launch of ChatGPT in late 2022, AI has increasingly found its way into our everyday decision-making. In December 2024, ChatGPT had 300 million weekly active users and over one billion queries per day (Singh, 2025). While it is unclear how many of the daily queries are related to a decision to be made, even if it is only a small fraction, it would still be an impressive number. In any case, it is hardly controversial that many people consult ChatGPT when making decisions, be it what to cook with ingredients X, Y, and Z, where to invest one’s money, or whether to leave one’s partner. Importantly, preliminary evidence suggests that ChatGPT’s advice can be as influential in people’s decision-making as advice from a human expert (Ikeda, 2024; Krügel et al., 2023). Taken together, it would not be surprising if ChatGPT became the world’s greatest decision-making assistant, both in terms of frequency of use and impact.

Of course, the use of AI as a decision-making assistant predates ChatGPT. For example, for more than 15 years, AI has been used in justice systems to predict convicts’ risk of reoffending or to forecast potential criminal activity, assisting courts and the police in their decisions (Meijer & Wessels, 2019; Travaini et al., 2022). AI also has a long history in medicine, where it supports clinicians in detecting diseases and making predictions about individual patient outcomes, which, in turn, influence clinicians’ recommendations and ultimately patients’ decisions (Kaul et al., 2020). But while the potential consequences of these AI-assisted decisions are significant, few people actually interact with these AI assistants, leaving them largely absent from our everyday life. At the same time, there are AI assistants such as Siri and Alexa that were present in many people’s lives even before ChatGPT. While these digital assistants can answer simple questions, they are primarily useful for voice-controlled device operations and performing basic tasks on devices. This is why their role as decision-making assistants is rather limited and likely restricted to choices with minor consequences, such as deciding which movie to watch or which restaurant to go to.

ChatGPT and LLMs in general appear to be different in this regard: you can ask them almost anything and usually get an answer that seems prima facie reasonable. So, if you have a question regarding a decision or need advice, you can simply ask an LLM. This is what makes them the perfect decision-making assistant—at least at first glance. Whether LLMs and other AI systems can effectively fulfill their role as useful decision-making assistants from a normative decision-theoretic perspective depends on their ability to help us not just in decision-making, but in *rational* decision-making. This leads to the following question: In what ways and to what extent can AI assist us in rational decision-making?

The present paper first examines the general ways in which AI can assist rational agents in their decision-making processes and then analyzes its usefulness for three types of decisions: easy choices, where each option is either more, less, or equally preferred to another option; hard choices, where the top-ranked options are on a par; and transformative choices, where we ourselves cannot know the expected value of each option. In easy choices, AI allows us to perform the decision-making process more efficiently and accurately, both of which increase the average value we derive from our choices. In hard choices, AI can assist us when committing to an option by supporting the process of creating new will-based reasons. In transformative choices, AI is unable to overcome the epistemic and metaphysical challenge present in such choices and thus does not enable rational decision-making. Overall, the analysis shows that while AI can usefully assist rational decision-making, its potential for predicting values and preferences is limited due to the inexistence of relevant data.

To make three assumptions underlying this paper explicit upfront: (1) This paper is about how AI can assist rational agents in their decision-making process. It is not about how AI assistance can turn non-rational agents into (more) rational agents. (2) This paper neglects the biases and other inaccuracies that are often present in AI systems (Ntoutsi et al., 2020; Srinivasan & Chander, 2021; Varsha, 2023). Accordingly, it assumes that AI operates under ideal conditions, with the result that if the relevant data is available, AI will draw correct conclusions from it. (3) Of course, these conclusions are not necessarily completely accurate, but the paper assumes that agents using AI can approximately judge how accurate they are.

The remainder of this paper is structured as follows: Sect.  2 recapitulates what is needed to make a rational decision. Section  3 analyzes whether and how AI can assist agents in rational decision-making, with its subsections focusing on easy choices, hard choices, and transformative choices. Section  4 concludes the paper.

## What is needed to make a rational decision?

Expected utility theory is the prevailing normative standard for rational decision-making (cf. Briggs, 2023; Buchak, 2022).^1^ It requires the agent to choose (one of) the option(s) with the highest expected value. Since this paper examines whether AI can assist agents in the process of making a rational decision, it employs expected utility theory as a deliberative tool (as opposed to merely an evaluative one). Such a deliberative approach to expected utility theory typically adopts a realist interpretation of decision theory, according to which credences and utilities are psychologically real and foundational to the preference ordering (Pettigrew, 2020). Accordingly, it is the agent’s credences and utilities that determine their preference ordering, which in turn determines their rational choice.^2^

To detect which option is rational to choose, the agent uses the following procedure: First, they take one of the available options. Second, they consider all possible outcomes this option can lead to and assess how much value they would derive from each of these outcomes. Third, they assess how likely each of these outcomes is. Fourth, they multiply every outcome’s value with its probability and add up all the products which results in the expected value of the option. Fifth, they do the same with every other option and rank the options in terms of how much expected value they provide. A rational agent then chooses the top-ranked option and, in this way, the option with the highest expected value. If more than one option is top ranked, they can randomly pick one of them, as each of these options maximizes expected value. Importantly, ordinary reasoners do not need to perform this procedure in a perfect manner to make a rational choice. A certain amount of approximation, uncertainty, ignorance, and mistaken beliefs is allowed. Otherwise, the normative standard for rational decision-making would be unattainable for ordinary reasoners (cf. Paul 2015b).

Taken together, to make a rational decision we need (justified) beliefs about: (1) what our options are; (2) which outcomes each option can lead to; (3) how probable these outcomes are; and (4) how much value these outcomes yield. Based on these beliefs we can calculate the expected value of all available options and choose (one of) the option(s) with the highest expected value.

## How can AI be helpful in rational decision-making?

There are two general ways in which AI can be helpful in obtaining the information required for a rational choice. The first concerns the mere collection of information necessary for the various assessments that are part of the decision-making process. To begin with, AI can help you identify more of the available options. Let’s assume you want to go out for dinner in a new city. An AI system can, for example, list all the available restaurants within a ten-minute walking distance. Next, let’s say that one of these restaurants specializes in shellfish dishes. AI can help you identify more of the possible outcomes that choosing to go to this restaurant can result in. For example, by use of AI, you may learn that eating shellfish can not only lead to various food experiences—from delicious to disgusting—but can also cause an allergic reaction. In a next step, AI can help you to find sources that indicate how likely the outcome of an allergic reaction is when eating shellfish, assuming that you do not already know whether you are allergic. The likelihood of this outcome seems to be roughly 3% (Warren et al., 2019) and should therefore be taken into account when deciding whether to eat shellfish. Finally, if you are unsure what an allergic reaction to shellfish is like, AI may help you to find descriptions or testimonies of it, which can then serve as a basis for determining the value of this outcome.^3^

The essential aspect of this use of AI for rational decision-making is that AI is used to find existing information—and not to create new information. Therefore, the information could also be found without AI; however, it is easier to search for it with AI. For decades, Google has been the search engine par excellence and the verb “to google” has even become a colloquial expression for searching something on the Internet. But with the advent of LLMs that can search the Internet in real time, this may change, as they provide remarkable results when searching for specific information and sources (Ulanoff, 2024).

The second general way in which AI can assist agents in rational decision-making is by generating new information. Here, we can differentiate three types of information that AI can produce. The first is useful for forming the beliefs necessary to determine the variables underlying our preference ranking. Often, AI-generated information makes a prediction about how likely some outcome is. To resume the example mentioned in the introduction, AI is used in justice systems to inform decision-makers about the probability of the outcome “recidivism” if they choose the option “release captive X.” The basis for such predictions is historical criminal justice data, including information on convictions and convicts on the one hand and recidivism rates on the other (Wang et al., 2023). In a similar manner, AI can help agents assess how much value they will derive from an outcome, provided that the relevant data is available.^4^ Finally, AI can also generate information about available options or possible outcomes that were not previously identifiable. For example, in a choice situation, an LLM may suggest that there are additional options to those identifiable by humans with the available information—something we currently see in drug development (Zhang et al., 2025). Similarly, with respect to an option, an LLM may suggest that there are additional possible outcomes to those identifiable by humans with the available information.^5^

Predictions of preferences are the second type of information that AI can generate. Predicting one’s preferences is equivalent to predicting which option is rational to choose. The agent does not need to calculate and compare the expected values of the available options but directly learns which option presumably has the highest expected value. AI systems that predict preferences can either do so based on data that already includes all the information necessary to make such predictions or they can infer preferences from people’s behavior/responses—at least that is the promise of the revealed preferences theory (Choi et al., 2014; Kreps, 1988; Varian, 1982). The latter approach is applied in several machine learning techniques, such as inverse reinforcement learning (Hadfield-Menell et al., 2016; Ng & Russell, 2000), reinforcement learning from human feedback (Ouyang et al., 2022), and direct preference optimization (Rafailov et al., 2024). Furthermore, recommender systems, which often model recommendation as the problem of showing users the items they are most likely to engage with, typically operate under the assumption that these items are also the most preferred ones (Hill et al., 2017; McInerney et al., 2018; Thorburn et al., 2022). For example, streaming platforms use recommender systems that are informed by data collected about the users’ profile and choices, particularly their past choices on the platform (Bischoff, 2023).

The third type of information that AI can generate is text that gains informative value through interaction with the agent. Specifically, by interacting with an AI system, an agent can start a process of reflection that ultimately leads to new insights. These insights are not generated by the AI itself, but the system plays a crucial role in bringing them about by prompting the agent’s thought process and guiding their exploration of ideas. For example, by interacting with an LLM, an agent who is torn between option A and option B may reflect on what they truly want and realize that option A satisfies their needs better than option B. To some extent, this may be somewhat similar to a conversation with a friend or perhaps also a coach about a decision one is struggling with.

So far, we have examined the ways in which AI can help us inform our beliefs and determine our preferences from a general decision-making perspective. What follows is a more detailed analysis of the extent to which the information AI can find and generate is useful for rational decision-making in easy choices, hard choices, and transformative choices.

### Easy choices

In an easy choice—with or without AI assistance—we can, in principle, rank all available options regarding the expected value they yield and choose the option with the highest expected value or pick one of the options with the highest expected value. Consequently, the rational decision-making procedure outlined in Sect. 2 can be performed without any obstacles (although a certain amount of reflection may be necessary along the way).

The main advantages of AI in easy choices are efficiency and accuracy. Let’s start with efficiency. Searching information with AI is much faster than searching information by ourselves. Within seconds, it can list a selection of options we have in a choice situation or sources that answer a question we asked. Of course, it is prima facie unclear whether the AI-suggested options and sources are the best ones out there. Maybe there is an option with an even higher expected value than the ones on the AI-generated list. Does this imply that taking the AI-suggested options as our choice set is not rational if we do not know whether the choice set actually includes the option with the highest expected value? Not necessarily since it is rational to optimize our search for options. If we had to search for options until we found the option with the highest expected value, we would typically need to find all the options. This is because we do not know whether an unconsidered option has a higher expected value than the options we have already considered. Now, optimizing involves stopping our search for a better option when the expected value of choosing the best option found so far exceeds the expected value of continuing the search and potentially discovering an even better option (Schmidtz, 2004). Accordingly, restricting ourselves to the options suggested by an AI system and choosing the one with the highest expected value among them can be equivalent to optimizing and therefore rational.

The same reasoning can be applied to searching for sources. Let’s resume the example of shellfish allergy. To estimate the probability of having an allergic reaction after eating shellfish for the first time, you can use AI to search for studies that examined the prevalence of shellfish allergy. It is likely that the AI system does not list all the studies on the issue but only a selection. Nevertheless, already this selection can be sufficient to form a justified belief about the probability of having an allergic reaction after eating shellfish for the first time. Put differently, you do not need to become a shellfish allergy prevalence expert before making a rational decision on whether to eat shellfish for the first time. The cost of doing so would not be justified by the gain in expected value achieved through making a better-informed decision (cf. Steele & Stefánsson, 2015).

An even more efficient way to make a decision is to use AI to directly predict one’s preferences. As in the case of an AI-assisted search for options, an AI system that predicts your preferences usually lists several possible options. But unlike in the former case, these options should already be aligned with your values and ranked in this regard. For example, Netflix has thousands of movies in its library. So, if you want to watch a movie on Netflix, it is virtually impossible to consider all the available options and then determine which one has the highest expected value. The Netflix algorithm helps you with your choice by suggesting movies you should like based on your prior Netflix choices: it uses a percentage to indicate how close the match is predicted to be for your specific profile.^6^ Whether the top-recommended movie truly has the highest expected value of all the movies on Netflix is doubtful. But it is still rational to go along with the recommended movie when doing so is equivalent to optimizing: it is better to watch a movie that is good, if not very good, now than to search for an even better one without knowing how long it will take to find it. And since the algorithm preselects movies that presumably match your preferences, the sequence of options you consider while optimizing is no longer random. This makes it likely that you will need to spend less time—and therefore less cost—finding a movie that is as good as the one you would have chosen if your optimization process were not influenced by a recommender system.

However, optimizing combined with the prediction of preferences has a problematic aspect. As Sharadin (2023) points out, the accuracy of a preference prediction algorithm cannot only be increased by better anticipating people’s given preferences but also by changing their preferences in such a way that they better match the prediction. Let’s say you watch a Netflix recommendation and enjoy it moderately. Netflix “notices” that you may not enjoy the movie too much, for example because you pause it several times. After you watched the movie, the algorithm could therefore update its prediction of your preferences and suggest another type of movie. Or it could stick to its prediction and suggest a similar type of movie, implicitly following the assumption that the more movies of this type you see, the more you will like them. As the mere exposure effect—the psychological phenomenon where repeated exposure to a stimulus increases an individual’s preference for it (Bornstein & Craver-Lemley, 2022)—demonstrates, this assumption has empirical support. Now, since you are optimizing, you are likely to choose one of the recommended movies even if it does not (yet) match your preferences very well. But the more you watch the recommended movies as a result of optimizing, the more you start to like them: the recommender system aligned your preferences to its predictions.

The idea of optimizing—be it with or without AI assistance—also comes with an epistemic challenge: When does the potential gain from finding an even better option no longer outweigh the cost of continuing the search? To answer this question, one needs to be able to estimate the expected value of continuing the search; and to do this, one needs to be able to estimate roughly what the expected values of the options one is searching for are, and how likely it is that one will find them in such and such a time. These are variables that we usually do not know. Thus, optimizing is not as straightforward as it may seem. Still, there is an approximative solution to this problem. By varying the amount of time you spend searching for a better and better option, you can learn about the approximate optimizing threshold for ending our search in different decision situations. For example, if you realize that, most of the time, your extensive search for a movie on Netflix still leads you to choose one of the recommended options or that the additional options you find are not really better than the recommended ones, you apparently optimize your Netflix choices by selecting a recommended movie right away.

Let’s continue with accuracy. AI-generated predictions can increase the accuracy of our beliefs based on which we make a rational choice. If you are wondering whether you should take an umbrella with you, you can take a look to the sky, trust some weather proverb, or consult a weather app. Most of the time, if you form your beliefs about whether it is going to rain based on an AI-powered weather forecast, it will be more accurate than the other two methods, leading to better choices: It is less likely that you will bring an umbrella even though it is not going to rain and/or that you will not bring an umbrella even though it is going to rain. The average value you derive from your choice increases. This is true for all predictions of how likely possible outcomes are. The better we are at accurately predicting these probabilities, the less mistaken beliefs lead to suboptimal decisions. Therefore, when AI helps us to form better beliefs about the probabilities of possible outcomes, it directly helps us to make better decisions, resulting in higher derived average value.

If AI detects possible outcomes that would be undetectable without it, or reveals additional options that would otherwise go unnoticed, it decreases what the decision-theoretic literature refers to as *unawareness* (cf. Steele & Stefánsson, 2015, 2021). Regardless of whether we are aware or unaware of such unawareness—that is, whether we know or not know that there are options or outcomes of which we are ignorant—reducing it increases the accuracy of our decisions. On the one hand, knowing more of an option’s possible outcomes allows us to better anticipate the option’s expected value and thereby to make a better-informed decision. On the other hand, having a more complete set of all possible options to choose from increases the probability that the option with the highest expected value in absolute terms is part of it. This is also true in the case of optimizing agents, provided that the search process starts with the options that are predicted to rank highest in our preference ordering. If there is no such preordering of options, the likelihood of ending up with an option that has lower expected value than the one you would have chosen without the additional options discovered by AI can also increase. This is the case if the average expected value of the newly discovered options is lower than the average expected value of the options which were already part of the choice set.

An AI-generated prediction of preferences can make our choices more accurate too. For example, if a recommender system has truly learned our movie preferences—at least approximately—the movies it recommends should have a relatively high expected value. Knowing this reduces some of the uncertainty about whether we will actually enjoy a movie: If we have not yet seen a movie, we do not know the exact value it will provide—only the range of possible values it could provide. An accurate Netflix algorithm that predicts that the movie is, say, a 95% match for our specific profile helps to narrow this range (or allows us to assign a more left-skewed probability distribution over this range). Of course, it is also possible to narrow this range without the assistance of AI.^7^ Still, an AI-generated prediction of preferences offers an additional reason to expect that we will like or dislike an unknown movie. It must be highlighted, however, that predicting preferences becomes more challenging the less similar the options are: while predicting preferences over “watching movie X” vs. “watching movie Y” is relatively easy, it is already more difficult for “watching movie X” vs. “seeing play Z”, and likely even more difficult for “watching movie X” vs. “cleaning the bathroom”. This is because there are fewer predefined choice sets with dissimilar options that allow an AI system to infer our preferences over these dissimilar options from our behavior. Thus, in the case of choice sets with dissimilar options, skipping the assessment of expected values and directly predicting preferences becomes more challenging.

Finally, as outlined in the previous section, AI systems are not only finders or generators of information but can also serve as a partner for reflection. In easy choices, having such a partner for reflection is particularly useful when one is not yet sure about how much value an option provides. Importantly, the usefulness of AI in this context is not that it predicts the expected value we will derive from the outcome but that it assists us in gaining clarity about this value ourselves. For example, an AI system might present thought-provoking questions and provide alternative perspectives that we might not have considered. This process then helps us to more accurately determine an outcome’s value and, consequently, make a more informed decision that better maximizes our expected value. So, rather than simply relying on the AI system to tell us an outcome’s value or the best choice, the interaction encourages self-reflection, empowering us to better understand our own values in the decision-making process.

To summarize, the use of AI in easy choices increases efficiency and accuracy. By finding information relevant for making a rational decision more quickly and ranking available options in terms of their preferability, the whole decision-making process becomes more efficient. By informing our beliefs with information that is more accurate than information collected or generated without AI, and by assisting us in reflecting on our values and thereby gaining more clarity on what our values actually are, the whole decision-making process becomes more accurate.

### Hard choices

Let’s say an AI system assisted you in your decision-making process, found and generated relevant information, and helped you reflect on your values. Yet, you do not know whether to choose A or B. Neither of the options is better than the other, but at the same time, they are also not equally good, meaning they yield exactly the same expected value; you are not indifferent between the two options and can simply pick one of them. As a result, none of the trichotomy of comparative relations—more preferred, less preferred, and equally preferred—applies between the two options. A ranking of the options regarding their preferability does not seem possible. Such a decision situation is called a hard choice.

The literature holds four prominent explanations for hard choices. First, some choices are hard because we are ignorant of some factors that are relevant to making the choice (e.g., Villiger, 2022). Thus, one of the three comparative relations does apply between the options, we simply do not know which one. Second, hard choices are difficult because they involve borderline cases of vague predicates (e.g., Broome, 1997; Constantinescu, 2016; Elson, 2014). This perspective is often paired with supervaluationism, which holds that, in such cases, each comparative relation is neither true nor false, while their disjunction is true (Flanigan & Halstead, 2018). Third, not all values are comparable with each other and options whose values are incomparable cannot be ranked. This is what makes choices that include such options hard (e.g., Anderson, 1997; De Sousa, 1974; Raz, 1986). Fourth, hard choices exemplify situations where the standard trichotomy of comparative relations fails, necessitating the introduction of a fourth comparative relation (e.g., Chang, 2002, 2012, 2017; Griffin, 1986; Parfit, 1984).

This paper’s analysis of whether AI can assist agents in hard choices is limited to the first and fourth explanations, with the latter discussed in this section and the former in the next. Specifically, this section examines whether AI can be useful in hard choices where there is *parity* between options. According to Chang (2002, 2017), an option cannot only be better than, worse than, or equally good/bad as another option. It can also be *on a par* with another option. Two options are on a par if we can assess their expected value and these values are comparable, yet none of the trichotomy of comparative relations applies. The options fall within the same neighborhood of value in terms of how much we (should) care about them, while differing significantly in the kind of value they represent, making indifference between the options an inadequate response. In more precise terms, there is an unbiased difference between the options (i.e., they differ in their properties while none is overall better than the other) with non-zero magnitude (i.e., the difference is significant enough to matter for the choice).

If options are on a par, the given reasons do not determine which one to choose. So, how do we choose then? Chang (2017) proposes two possibilities: committing and drifting. Committing involves exercising our normative power to generate new will-based reasons for favoring one option over another by endorsing a specific feature of that option through our agency. By committing to an option, we add additional value to this option and therefore become the author of our own rational decision (Goodman, 2021). In contrast, drifting involves intentionally choosing an option, but doing so in a noncommittal way, that is, without endorsing or aligning ourselves with any particular feature of the option.

The use of AI for drifting is limited because a drifter can simply choose one of the options. Still, some agents may prefer that the AI system suggests which option they should choose because they are unable or do not want to make that choice themselves. In that case, AI can play the role of a coin toss.

The situation is different in the case of committing. Since committing means to create new will-based reasons for preferring one option over the other(s), AI can assist us in this creation process. Specifically, there are two ways AI can be useful here. First, we chat with an LLM and for example discuss the different features of our options. This conversation may then prompt new will-based reasons by initiating reflection processes. It is important to notice that in such a case, the content of the will-based reason is created entirely by us. Second, we chat with an LLM and identify a non-given reason (i.e., a reason that is not already contributing to the value of the option that led to parity) in its produced text and adopt it. Thus, in this second case, the content of the will-based reason is at least partly AI-created.

To give an example, let’s say you must choose between becoming a physician or becoming a philosopher. These two options seem to differ significantly in the kind of value they represent: we may say that while becoming a physician represents the value of helping others, becoming a philosopher represents the value of pursuing truth and understanding. Suppose that neither of these values dominates the other, nor should we be indifferent between them (nor do other values relevant for the choice make one option better than the other). In this case, the two options are on a par. Now, if you want to commit to one of them, you must generate will-based reasons. Specifically, you must generate reasons why *you personally* want to follow either of these values. This is where AI may assist you. For instance, your interaction with AI may lead you to reason that you want to become the kind of person who takes responsibility for others’ well-being, thus committing to becoming a physician. Or you may commit to becoming a philosopher as you identify and adopt the reason from an AI text that you want to become someone who pursues truth regardless of practical utility, choosing to prioritize that aspiration.

It might be objected that if we use AI when committing—especially when we adopt AI-produced non-given reasons—we transfer the authorship of our own rational decision to the AI system and consequently miss the whole point of what it means to commit in a hard choice. This objection is not unfounded. To delegate our hard choice to an AI system certainly seems to be problematic in terms of agency. Let’s go back to the choice between becoming a physician or becoming a philosopher. It would be inappropriate to ask ChatGPT what to do and, assuming it tells you why one option is better for you than the other, simply commit to what it says and choose the corresponding option. Such commitment would not be grounded in your will-based reasons. In fact, such commitment would not be grounded in any reasons. As many authors argue, LLMs can only produce text, not reasons, because they do not think (cf. Starke & Jox, 2024; Stoljar & Zhang, 2024). This is why simply committing to what LLMs say would result in an action that is not grounded in reason. However, the text that LLMs produce can become a reason if we identify it as such. In the case of committing in a hard choice, we must identify a non-given reason within the text. If we recognize such a non-given reason, we can adopt it and thereby transform it into a will-based reason. Ultimately, we may then commit to this will-based reason and choose accordingly.

Porsdam Mann et al. (2024) argue along similar lines regarding the role of LLMs in medical co-reasoning. According to them, the fact that “reasons” are originally generated by an LLM rather than a doctor or patient should be irrelevant. What truly matters is that humans can reflectively evaluate the input provided by the LLM to reach an all-things-considered conclusion. That is why including LLMs in medical co-reasoning is not problematic per se and can be beneficial given that LLMs help to expand the set of reasons. Accordingly, if it is our ability to identify and endorse a reason that makes the reason our own, AI can play a useful role in helping us commit to an option in the context of a hard choice.

As an aside, the existence of parity also poses a challenge to AI (Dobbe et al., 2021; Goodman, 2021). On the one hand, cases of parity cannot be solved through mathematical formalism as there needs to be an intentional choice by an agent, posing a problem for AI-substituted decision-making (but not for AI-assisted decision-making). On the other hand, it is almost impossible to infer cases of parity from behavioral data, which is relevant for AI learning techniques that try to infer human preferences from human behavior. If an agent commits to an option, their choice behavior cannot be differentiated from a situation where they prefer the option. For instance, if you commit to playing tennis when it is on a par with playing soccer, you will choose tennis over soccer the next time too, just as if you had always preferred tennis. Similarly, if an agent drifts into an option, their choice behavior cannot be differentiated from a situation where they are indifferent between options. If you drift into playing tennis when it is on a par with playing soccer, you may choose soccer over tennis the next time, just as if you were indifferent between the two.

The problem is that if AI conflates choices where the top-ranked options are on a par with easy choices, its inability to discriminate between the two leads it to overlook situations in which we should become the authors of our own decisions. This restricts our autonomy in rational decision-making. For instance, AI may suggest that we are indifferent between two options, even though we could instead commit to one of them and thereby make it the preferable option for ourselves. Conversely, AI may suggest that one option is better than the other, even though this is actually a matter of commitment, ignoring the possibility that we could commit to (or drift toward) either option. Importantly, cases of parity typically arise in significant, life-altering choices—such as the choice between two careers—precisely because such choices tend to involve conflicts between fundamental values. This is why it is crucial for AI systems to take hard choices into account, as they would otherwise fail precisely when their assistance is most relevant.

In the end, to prevent AI from mistaking hard choices for easy choices, it seems necessary to directly program it to recognize (potential) cases of parity in certain value constellations. One possible approach, which can only be briefly sketched in this paper, is to let AI identify parity through structural features of the value space, including contexts where: (a) values differ in kind rather than in degree; (b) neither value dominates the other across relevant evaluative dimensions; and (c) the data is consistent with multiple, equally rationalizing explanations. If an AI system were programmed to detect such configurations, it could flag potential cases of parity whenever these structural features appear.

### Transformative choices

A ranking of options in terms of their preferability can also be incomplete within the trichotomy of comparative relations because it is not possible to assess the expected value of every option. This is the case in so-called transformative choices. Paul (2014) argues that if we have never experienced the experiential kind to which an outcome belongs, then experiencing the outcome for the first time leads to an *epistemic transformation*: we learn what experiencing this kind of outcome is like and thereby its subjective value. The subjective value, which contributes to an outcome’s total value, is the value of *living* an outcome. Importantly, while there are some exceptions, the subjective value of an outcome that belongs to an unfamiliar experiential kind can only be learned through experience. This leads to the following problem: if we can only learn an outcome’s subjective value through experiencing it, we remain ignorant of the outcome’s expected value before and during the first time we choose it. As a result, we can neither rationally choose nor decline an option with an epistemically transformative outcome. To take a prominent example in the literature: You only know what it is like to be a parent by becoming one. Therefore, when you choose (or decline) to become a parent, you do not know the expected value of this option. It is epistemically inaccessible and only gets accessible through undergoing the experience of becoming a parent. This is why standard decision theory can neither tell us whether it is rational to become a parent nor whether it is rational not to become a parent.

Experiencing an outcome can transform us not only epistemically but also personally. A *personal transformation* occurs when an experience changes what we value. For instance, becoming a parent not only teaches you what it is like to be a parent but may also shift your values: As a non-parent, you might have valued going out and partying, while as a parent, you may come to value staying at home with your family or having a game night. The possibility of personal transformation in transformative choices further complicates rational decision-making: not only do you not know the subjective value of a transformative outcome, but you also do not know if and how it will change your other values. In addition, decision theory does not tell you how much weight your future preferences should have compared to your present preferences. Rational choice seems to have broken down completely.

Several authors have suggested ways for rational decision-making in transformative choices (e.g., Dougherty et al., 2015; Kauppinen, 2015; Pettigrew, 2015, 2016, 2019; Reuter & Messerli, 2018; Sharadin, 2015; Villiger, 2021, 2023). One of them is to consult testimony of those who have already experienced the transformative outcome and, on that basis, assess its expected value. For example, if you are considering whether to become a parent, you should talk to parents and perhaps review empirical studies about the effects of parenthood on life satisfaction. The information you gather will then allow you to assess the expected value of becoming a parent.

This seems like an ideal use case for AI-assisted rational decision-making: instead of gathering the information and making a value prediction ourselves, we use an AI system to do it for us. However, there is a major challenge at the beginning of any value prediction, with or without AI: do we have the necessary data to make such a prediction?

To highlight the severity of this challenge, let’s first look at predicting probabilities. Typically, you have a data set (e.g., historical criminal justice data) with input variables (e.g., information on convictions and convicts) and an output variable (e.g., the recidivism rate of the convicts). Using a technique such as supervised learning, the algorithm learns to predict the output variable from the input variables. The prediction is a number between 0 and 1, which represents the probability of the outcome and is therefore easy to interpret.

This is quite different when predicting values. In order to predict values, we need a data set that includes the desired output variable, namely the value of outcome X. If we follow the framework of expected utility theory, a numerical utility value represents the value of outcome X. The problem with utility values is that they are defined on an interval scale. Therefore, knowing how much utility other people derived from an outcome by itself is completely useless. Only if there are at least two “utility value anchors”, which allow us to determine the relative positions and intervals of the utility values of these other people and our utility values, the number becomes meaningful (Isaacs, 2020). Put differently, we would need to be able to link two of our utility values with two of the data, allowing a translation between the utility value scales. The problem is that these utility value anchors that would enable such a translation seem not to exist.

There is a less demanding method to indicate the value of an outcome: verbal ratings. For example, an outcome can be rated as extremely unliked, unliked, rather unliked, neither unliked nor liked, rather liked, liked, or extremely liked. These verbal ratings can then also be converted into a numerical scale, where 1 stands for extremely unliked and 7 for extremely liked. Provided that a data set includes such ratings about an outcome X along with the profiles of those who made these ratings, it becomes possible to make a clear prediction about how much someone with profile Y will like the outcome X. However, this prediction has its own problems. First, it is unclear whether the intervals between the seven possible ratings truly have the same distance—something we implicitly assume when we number them from 1 to 7. Second, it is unclear whether the people whose data is used for the prediction have on average the same understanding of the verbal evaluations as we do. Third, the likeability of an outcome is not the only factor that contributes to its value— and the direct use of the term “value” in verbal ratings is not really an option because it is very abstract. Taken together, while verbal ratings are easier to interpret and more feasible than utility values, they introduce many imprecisions.

Another approach for rational decision-making in transformative choices is to skip the prediction of values and directly predict preferences. The advantage of predicting preferences is that they can be inferred from behavior (if we follow revealed preferences theory). For example, the Netflix algorithm does not “know” how much we value the movies in the library, but it can still infer our preferences from our behavior on the platform. These inferred preferences are, of course, not independent of our values. Still, the values can be neglected because, ultimately, the algorithm’s success rate is not determined by whether it accurately predicts how much we value an option, but rather by whether we choose and watch the suggested option. However, as we already know, there are three problems with predicting preferences: First, AI may align our preferences to its predictions (rather than the other way around); second, the less similar the available options are, the more difficult it becomes to predict preferences; and third, AI can hardly infer cases of parity from behavior. Now, in the context of transformative choices, an even more severe problem arises: since people do not know which option maximizes expected value in such choices, inferring preferences from behavior becomes pointless. As a result, ignoring values and directly predicting preferences is not a promising approach for AI-assisted rational decision-making in transformative choices.

Let us now put aside the question of whether we have the relevant data to make value predictions and assume that we can predict the expected value of a transformative option. Paul (2015a) would still criticize such value predictions because they reveal an outcome’s value not from the perspective of the individual making the choice, but from the perspective of the average member of the population on which the prediction is based. Additionally, these predicted values do not differentiate between the future value for the agent as they are now and the future value for the agent after a potential personal transformation. Taken together, the agent is alienated from a transformative decision that is based on third-personal information in two ways. First, they are disconnected from the values guiding the decision, as these values are not their own. Second, they are alienated from the person they might become by choosing a transformative option—something mere consideration of values cannot capture. Paul calls the first type of alienation epistemic alienation and the second type metaphysical alienation. Can AI be useful in solving these two challenges that transformative choices pose to rational decision-making?

We start with the question of whether AI can help us to assess the expected value that *we* derive from a transformative outcome and thereby reduce epistemic alienation. The idea is that even though we are unable to anticipate our expected value of an outcome, AI can predict it. A recent discussion in bioethics has touched on this idea. Earp et al. (2024) argue that if a patient is incapacitated and a substituted medical decision is necessary, a personalized patient preference predictor should co-inform surrogates decision-making. The concept of a patient preference predictor (without the *personalized*) is already a decade old and has the purpose to predict what a patient would choose in a given situation based on the preferences of similar individuals (Rid & Wendler, 2014). To do so, it leverages demographic, clinical, and psychosocial data to generate group-specific predictions. Put differently, the patient preference predictor considers testimony only of people who are relevantly similar to the patient—so similar that their preferences are assumed to largely align with those of the patient. Now, Earp et al. (2024) want to take this a step further: the *personalized* patient preference predictor (4P) not only uses population data that allow the identification of fine-grained reference classes but also personal data from the patient, such as emails, blog posts, or social media posts. This way, the 4P makes predictions based on what the patient wants, as expressed in their data, and not on what people who are similar to the patient want—at least that is the promise.

It has already been suggested that the 4P should not only be used for surrogate decision-making but also to infer what is in the best interest of incapacitated patients (Berger, 2024). From here, it is not far to argue that if capacitated patients are unsure about which treatment is in their best interest, they could consult the 4P (cf. Starke & Jox, 2024). Schwan (2024) even believes that the 4P might “help justify overruling a capacitated patient’s decision when there is good reason to believe that their decision is sufficiently contrary to their more fundamental commitments” (p. 40). This would imply that the 4P has the potential to know the values of capacitated patients better than the patients themselves, even if patients say they know their values. Now, the outcomes of major medical decisions are often transformative (Hofmann, 2023; Villiger, 2025). Thus, if we assume that AI systems such as the 4P can predict people’s values in such choices, we should also assume that they can do so in transformative choices more generally. And since these AI systems form their prediction based on our own data, the AI-predicted values that guide our decision should be our own values, solving one challenge that transformative choices pose to rational decision-making. Is this line of argument convincing?

It is not. The way a P4 predicts our preferences is not truly based on our own values. It merely uses our personal data to better fine grain the reference class we belong to. In the end, the P4 still makes a generalization based on that reference class. Let’s go back to the decision to become a parent. If we use a P4 in this decision, it will not only consider what others who are relevantly similar to us had chosen in this decision situation and (if possible) how much value they derived from it, but also personal data of us. For example, it may consider how often we watch baby videos on Instagram and YouTube, what we wrote about parenthood in our digital diary, blog and social media posts, text messages, and emails, how many photos we have taken from the babies of our friends and siblings, and so on. However, this personal data alone is useless, as it is underdetermined for making predictions about our own values concerning parenthood. Only if we apply a generalization to the data, we can make a prediction. Such a generalization could for example be “the more someone watches baby videos on Instagram and YouTube, the more they will value parenthood.” Combining this generalization with the personal Instagram and YouTube data allows for a personalized prediction—but only in the sense that personal data has been used to make the prediction and not that the prediction is exclusively based on personal data.

Generalizations do not need to be derived from population data. If an agent has consistently chosen vanilla over strawberry ice cream whenever faced with the choice, we can generalize that they will always prefer vanilla over strawberry ice cream. Based on this, we can predict that the agent will also choose vanilla in the future. Then again, this kind of generalization would be useless for transformative decisions, since there are no past decisions that are roughly equivalent to transformative decisions (otherwise they would not be transformative). But what about the following case: whenever an agent wrote more positively about one option compared to the others in the digital sphere, the more value that option provided to the agent when choosing it. From this, we can generalize that the more positively an agent writes about one option compared to the others in the digital sphere, the higher its expected value. This generalization is based on personal data and could be applied by a P4 in a transformative choice to make a truly personalized prediction. Then again, basing one’s choice on such a generalization seems inappropriate: if we assume that the value of a transformative outcome is epistemically inaccessible, why should the extent to which we write positively about it be predictive of its value? In the end, while a prediction derived from such a generalization is based on some of our values, it is not based on our values concerning the transformative outcome. The prediction cannot tell us why we value the transformative outcome.

Taken together, the predicted values of a P4 that should guide us in making a rational transformative choice are not our own values of the transformative outcomes. If the values concern the transformative outcomes, they are not our own but reflect the reference class to which the P4 assigns us; and if the values are derived from personal generalizations, these generalizations do not specifically concern the transformative outcomes. As a result, our own values of the transformative outcomes remain concealed. This, in fact, points to a dilemma when using AI in transformative decision-making more generally: either our AI-assisted choice does not build on our own values but on those that our reference class derived from the specific transformative outcome, or it does build on our own values but not on values concerning the specific transformative outcome. How should one decide if these two sources point in different directions?

It could be argued that whether the prediction is based on our own values of the transformative outcomes is ultimately irrelevant. The only thing that matters is whether the predicted values are accurate. So, let’s assume that the values a P4 predicts are indeed accurate. This would still leave the problem that the mere consideration of values does not tell us who will derive them: our current self or our transformed self. But of course, just as AI could predict the values of transformative outcomes, it could also predict whether and how our values will shift when we choose a transformative outcome. Yet even if we again assume that these predictions are accurate and we therefore know how our values will shift, we would still be alienated from our transformed self. Can AI somehow reduce this metaphysical alienation?

There is the idea of a digital twin or digital doppelgänger, for example by fine-tuning an LLM with personal data (Iglesias et al., 2024).^8^ Theoretically, we could create such a digital twin and have it choose a transformative option, such as becoming a parent, resulting in algorithmic changes that represent the value shifts caused by us becoming a parent. By chatting with our “digital twin plus parenthood”, we learn and get familiar with how becoming a parent will change us. This helps us to become less alienated from our parent-self.

Apart from the fact that all of this is far from being (momentarily) feasible if we want digital twins and transformed digital twins to be accurate, it is questionable whether interacting with our transformed digital twin can truly reduce alienation regarding our transformed self.^9^ In the end, we are neither interacting with our transformed self nor with someone who has experienced parenthood. Instead, we are interacting with something that mimics our transformed self and simulates having experienced parenthood. With this in mind, it is unclear why interacting with someone who has actually experienced parenthood and was relevantly similar to us before parenthood should be less effective in reducing metaphysical alienation than interacting with an AI system.

Finally, the question of how much weight should be given to current versus future preferences in transformative choices cannot be answered by AI, as it is a normative issue. Several accounts in the literature offer different answers to this question (e.g., Isaacs, 2020; Pettigrew, 2019; Schulz, 2020; Ullmann-Margalit, 2006), and it remains open which approach should be followed. Thus, even if (1) AI allows us to anticipate the values of transformative outcomes and the value shifts they can bring about, (2) AI enables us to become less metaphysically alienated from our transformed self, and (3) we neglect that the predictions the AI system makes are not based on our own values regarding the transformative outcome, the rational option to choose would still be undetermined in transformative choices.

## Conclusion

This paper analyzed in what ways and to what extent AI can assist agents in rational decision-making. It found that, from a general perspective, AI can help us find existing information or create new information, both of which can lead to better-informed beliefs about what options are available, what outcomes they can lead to, how likely those outcomes are, and how much value they provide. The extent to which this promotes rational decision-making depends on the type of choice an agent is confronted with. In easy choices, AI assistance makes rational decision-making more efficient by accelerating the optimization process and more accurate by reducing mistaken beliefs. In hard choices, AI assistance helps agents to create new will-based reasons when committing. It does so by either prompting reflection processes from which new will-based reasons arise or by producing text in which agents identify non-given reasons which they then adopt and thereby turn into new will-based reasons. Finally, in transformative choices, AI assistance does not enable rational decision-making. It can neither predict the value a transformative outcome will provide *us* nor (better) reduce the metaphysical alienation between our current self and the transformed self that we may become after undergoing the transformative experience. Taken together, we see that if the values of our options do not allow us to determine the rational choice in the absence of AI—whether because they are on a par or because we are ignorant about (some of) them—the presence of AI does not change that.

## Data Availability

Not applicable.

## Footnotes

1 Note, however, that expected utility theory is not an uncontroversial normative standard of rational choice (for rival theories, see Buchak, 2022).

2 This understanding of expected utility theory differs substantially from the evaluative-constructivist one, which is more common, especially in economics. On an evaluative constructivist understanding, (1) whether choosing a certain option is rational does not depend on our reasons for doing so but solely on whether it maximizes expected utility, and (2) the preference ordering is primary, meaning that it does not need to be justified any further (Pettigrew, 2020).

3 Obviously, the value is negative. However, you need to determine how negative it is to calculate the expected value of eating shellfish.

4 As will be shown in Sect. 3.3, the relevant data for value predictions is often inexistent.

5 One challenge here is the uncertainty about whether these newly identified options and outcomes truly exist as proposed. For example, while AI is used in drug development to identify new drugs, it is of course not omniscient in this regard. Thus, drugs designed and properties predicted by AI must still undergo validation through wet-lab experiments (Zhang et al., 2025).

6 Apparently, about 75% of what users watch on Netflix comes from its personalized recommendations (Antonyuk, 2024).

7 For example, you may know that you have enjoyed all of Director X’s movies so far, making it likely that you will also enjoy a movie by Director X that you have not yet seen. Or you may have noticed that you usually like movies that are well-rated on movie platforms, making it likely that you will also enjoy a well-rated movie that you have not yet seen.

8 For example, the AI chat platform *Future You* is based on the idea that users can explore different possibilities of their future, which should help them to find the one that feels most authentic and inspiring to them (Yee, 2024). The developers emphasize, however, that they are not actually—in the sense of accurately—predicting one’s future self.

9 Particularly in the case of parenthood, the idea that an AI system can emulate how this experience will transform us is far from feasible as this transformation also depends on the very child we become a parent of.
